# Supplementary material for: Assessment of Bioactive Compounds under Simulated Gastrointestinal Digestion of Bee Pollen and Bee Bread: Bioaccessibility and Antioxidant Activity
Source: Antioxidants (Basel). 2021 Apr 23;10(5):651. doi: 10.3390/antiox10050651 (PMC8146390; doi:10.3390/antiox10050651)
Supplement: Supplementary file 1 [file antioxidants-10-00651-s001.zip › antioxidants-1180355-supplementary.pdf]

Table S1. Preparation of stock solutions of simulated digestion fluids: simulated saliva fluid (SSF), gastric fluid (SGF) and intestinal fluid (SIF) stock solutions. The final volume for each digestive fluid is 500 mL and at a concentration of 1.25 × [1].

			SSF		SGF		SIF	
			pH 7		pH 3		pH 7	
Constituent	Stock conc.		Vol. of stock	Conc. in SSF	Vol. of stock	Conc. in SGF	Vol. of stock	Conc. in SIF
	**g/L**	**mol/L**	**mL**	**mmol/L**	**mL**	**mmol/L**	**mL**	**mmol/L**
KCl	37.3	0.5	15.1	15.1	6.9	6.9	6.8	6.8
$KH2PO_4$	68	0.5	3.7	3.7	0.9	0.9	0.8	0.8
$NaHCO_3$	84	1	6.8	13.6	12.5	25	42.5	85
NaCl	117	2	—	—	11.8	47.2	9.6	38.4
$MgCl_2(H_2O)_6$	30.5	0.15	0.5	0.15	0.4	0.1	1.1	0.33
$(NH_4)_2CO_3$	48	0.5	0.06	0.06	0.5	0.5	—	—
**For pH adjustment**								
	**mol/L**		**mL**	**mmol/L**	**mL**	**mmol/L**	**mL**	**mmol/L**
NaOH	1		—	—	—	—	—	—
HCl	6		0.09	1.1	1.3	15.6	0.7	8.4
**$CaCl_2(H_2O)_2$ is not added to the simulated digestion fuids**								
	**g/L**	**mmol/L**		**mmol/L**		**mmol/L**		**mmol/L**
$CaCl_2(H_2O)_2$	44.1	0.3		1.5*		0.15*		0.6*

* is the corresponding Ca^{2+} concentration in the final digestion mixture.

Table S2. Correlation analysis between phenolic content and antioxidant capacity.

Assays	r	Confidence interval of r	Significance level
**Between phenolic content and antioxidant capacity**			
TPC – DPPH	0.799	0.584 to 0.909	*
TPC – Reducing power	0.743	0.484 to 0.881	*
TFC – DPPH	0.784	0.557 to 0.902	*
TFC – Reducing power	0.562	0.419 to 0.862	*
**Between phenolic and flavonoid**			
TPC – TFC	0.873	0.724 to 0.943	*
**Between DPPH and reducing power**			
DPPH – Reducing power	0.839	0.658 to 0.928	*

Twelve paired average samples from each test were used in the comparison. r value represents the Pearson's linear correlation value. The level of significance was expressed as $*p < 0.01$.

References

1. Minekus, M.; Alminger, M.; Alvito, P.; Ballance, S.; Bohn, T.; Bourlieu, C.; Carriere, F.; Boutrou, R.; Corredig, M.; Dupont, D. A standardised static in vitro digestion method suitable for food–an international consensus. *Food Funct.* **2014**, *5*, 1113-1124.
